# Phenotypic Mismatches Reveal Escape from Arms-Race Coevolution

**DOI:** 10.1371/journal.pbio.0060060

**Published:** 2008-03-11

**Authors:** Charles T Hanifin, Edmund D Brodie, Edmund D Brodie

**Affiliations:** 1 Department of Biology, Utah State University, Logan, Utah, United States of America; 2 Department of Biology, University of Virginia, Charlottesville, Virginia, United States of America; Cornell University, United States of America

## Abstract

Because coevolution takes place across a broad scale of time and space, it is virtually impossible to understand its dynamics and trajectories by studying a single pair of interacting populations at one time. Comparing populations across a range of an interaction, especially for long-lived species, can provide insight into these features of coevolution by sampling across a diverse set of conditions and histories. We used measures of prey traits (tetrodotoxin toxicity in newts) and predator traits (tetrodotoxin resistance of snakes) to assess the degree of phenotypic mismatch across the range of their coevolutionary interaction. Geographic patterns of phenotypic exaggeration were similar in prey and predators, with most phenotypically elevated localities occurring along the central Oregon coast and central California. Contrary to expectations, however, these areas of elevated traits did not coincide with the most intense coevolutionary selection. Measures of functional trait mismatch revealed that over one-third of sampled localities were so mismatched that reciprocal selection could not occur given current trait distributions. Estimates of current locality-specific interaction selection gradients confirmed this interpretation. In every case of mismatch, predators were “ahead” of prey in the arms race; the converse escape of prey was never observed. The emergent pattern suggests a dynamic in which interacting species experience reciprocal selection that drives arms-race escalation of both prey and predator phenotypes at a subset of localities across the interaction. This coadaptation proceeds until the evolution of extreme phenotypes by predators, through genes of large effect, allows snakes to, at least temporarily, escape the arms race.

## Author Summary

Arms races between natural enemies can lead to the rapid evolution of extreme traits, high degrees of specialization, and the formation of new species. They also serve as the ecological model for the evolution of drug resistance by diseases and for host–pathogen interactions in general. Revealing who wins these arms races and how they do so is critical to our understanding of these processes. Capitalizing on the geographic mosaic of species interactions, we examined the dynamics of the arms race between snakes and their toxic newt prey. Garter snakes in some populations have evolved dramatic resistance to the tetrodotoxin defense of the their local prey. By evaluating the pattern of mismatches between toxicity and resistance, we discovered that predators sometimes escape the arms race through the evolution of extreme resistance, but that prey never come out ahead. The reason for this one-sided outcome appears to depend on the molecular genetic basis of resistance in snakes, wherein changes to a single amino acid residue can confer huge differences in resistance.

## Abstract 3

Who wins in the arms race between predators and prey? In the interaction between snakes and toxic newts, predators sometimes escape the arms race through the evolution of extreme resistance, but prey never come out ahead.

## Introduction

The process of coevolution plays out on a wide spatial and temporal stage [1]. At any point in space and time, we might observe a given pair of interacting populations occupying any position within the range of their coevolutionary trajectory: from the early stages of escalation, to equilibrium, to cost-induced descalation. A variety of historical, geographical, and ecological factors influence the condition of a set of interacting populations [1–7], rendering it exceedingly difficult to draw inferences about the coevolutionary process through the examination of one or a few localities at a single time [8,9]. Some forces and dynamics, such as gene flow or selection mosaics, can be effectively revealed in experimental systems with short generation times [10–12]. However, for natural populations of long-lived organisms, it is only by extending the analysis of coevolution across the geographic range of an interaction that we can begin to elucidate the dynamics and history of the process [9,13–25].

Coevolution is fundamentally driven by reciprocal selection that is generated by the ecological interactions of coexisting species [1,2,26]. The phenotypic interface of coevolution is defined as the set of traits that mediate these interactions [27,28]. When interacting species have roughly matched abilities at the phenotypic interface, the potential for strong reciprocal selection exists, because interactions between individuals of the two species are expected to have variable fitness consequences for one another [9,16,27,29]. At high levels of mismatch in abilities, however, variable fitness is not associated with variation in trait values for either species—all hosts may be resistant to infection by a parasite or all prey may be too toxic for any predator to ingest. In such cases, reciprocal selection no longer occurs, and the coevolutionary process for this pair of populations is suspended until other forces such as gene flow or mutation introduce new variants in one or the other population of interactants. By comparing the degree of functional mismatch among localities, we can infer which populations currently have the potential to experience reciprocal selection at the phenotypic interface of coevolution.

Defining and recognizing a phenotypic match is nontrivial. Matches are usually considered coevolutionary “hot spots,” where current reciprocal selection is strong [1,2,4,21,22,30–32]. Despite apparent matched levels of traits based on functional inference, two interacting populations may not experience reciprocal selection for any number of unidentified ecological or genetic reasons that mediate fitness (e.g., interactions with other species, energetic costs associated with the production of phenotypic interface traits, abiotic factors that obscure variance in fitness [1,2,7,17–20,33–35]). Thus, functional phenotypic matches may not necessarily reflect true coevolutionary hot spots.

Mismatches, on the other hand, should be more explicitly recognizable. By definition, mismatches are localities wherein the distributions of traits at the phenotypic interface are functionally nonoverlapping; the most extreme traits in one species generate no ecological effect on the most extreme trait of the other species. In this case, individuals of each species will not experience variable fitness outcomes (and thus no selection or evolutionary response) related to the traits in question. Mismatches are sometimes identified by differences in mean phenotypes [6,9,16,31,34,36,37]. However, because interacting individuals invariably encompass a range of phenotypes, the more critical requirement is to identify nonoverlapping phenotypic distributions around some critical performance threshold [27,29]. An evaluation of mismatches as “cold spots” therefore requires a distributional evaluation—if means differ but distributions can produce pairings of individuals with variable fitness outcomes (e.g., the least resistant predator coupled with the most toxic prey), reciprocal selection might still result.

Across the range of an ecological interaction, the degree of performance mismatch is expected to vary broadly. Once considered a general piece of evidence for coevolution, tightly matched phenotypes between natural enemies may be relatively uncommon among [4,6,36,38–40] and within species [35,37,41], as suggested by recent theory. So many factors influence whether traits are matched or not that inferences of underlying processes that are based on pattern alone are tenuous [1–6,24,39,40,42]. Regardless of which factors influenced current phenotypic patterns, the distribution of phenotypic mismatches can provide insights into the current selection dynamics and coevolutionary trajectories. Mixed patterns of phenotypic match and mismatch are observed in a wide variety of empirical systems including the following: host–parasite systems [24,25,38,43–45], plants and seed predators [9,14–16,18], and chemically mediated coevolution between parsnips and insect herbivores [21,22]. In each of these examples, phenotypic mismatches have revealed a geographically variable pattern of coevolutionary selection.

**Figure 1:**
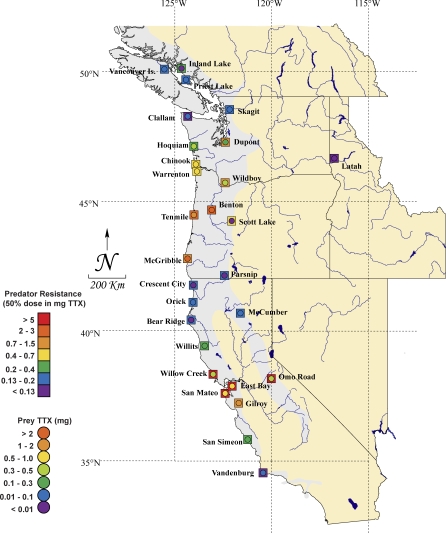
The Geographic Distribution of TTX Resistance of Garter Snakes and TTX Toxicity of Newts in Western North America Twenty-eight populations of a snake predator, T. sirtalis (squares), and co-occurring toxic prey, *Taricha* newts (circles) sampled throughout western North America are shown. Colors (denoted in legend) indicate TTX-resistance as the amount of TTX (mg) required to reduce an average adult female *Th*. *sirtalis* (at a given locality) to 50% of its baseline performance and toxin level as the total amount of TTX (mg) expected in the skin of an average adult *Taricha* newt. The range of the genus *Taricha* is shown in gray, but extends beyond the area shown in the map to the north along the coast of British Columbia into southeastern Alaska.

We examined variation in the phenotypic interface of the arms race between the garter snake predator, Thamnophis sirtalis, and its deadly prey (newts of the genus *Taricha*) to assess patterns of functional mismatch across both the geographic and phenotypic ranges of this interaction. Our analysis spans the geographic range of sympatry of *Taricha* and T. sirtalis, over 2,000 km from British Columbia, Canada, to southern California (Figure 1). The 28 localities sampled encompass the full variation of predator and prey phenotypes known in this interaction. We used a detailed functional model of the phenotypic interaction between newts and snakes to identify localities where trait distributions are sufficiently mismatched as to obviate current reciprocal selection. The emergent patterns of phenotypic escalation and phenotype mismatch reported here reveal not only spatially variable present day coevolution, but also suggest historical dynamics that cannot be observed in more geographically limited analyses. Mismatches invariably occur in the direction of predators exhibiting traits that are more extreme than necessary to exploit local prey and, consequently, not experiencing selection as a result of prey toxicity. Within localities that display trait mismatches, the level of phenotypic escalation indicates different coevolutionary histories across the range of populations.

### Background

The phenotypic interface of the predator–prey interaction between garter snakes and newts of the genus *Taricha* revolves around tetrodotoxin (TTX). TTX is one of the most potent neurotoxins known, binding to the outer pore of voltage-gated sodium channels in nerve and muscle tissue, thereby blocking the propagation of action potentials [46,47]. *Taricha* have high levels of TTX in the skin and are lethal to a variety of potential predators [28,48–52]; individuals from some populations have up to 14 mg of toxin, which is enough TTX to kill thousands of mice or up to 10–20 humans. A growing body of evidence suggests that newts produce their own TTX, but the genetics and biosynthesis of this process are poorly understood [50,53–57]. Some garter snakes of the genus *Thamnophis* have evolved resistance to this prey toxin through modifications of the sodium channel structure in skeletal muscle [58,59] and are capable of ingesting whole adult newts without permanent adverse effects [60,61]. Resistance in snakes is heritable [62,63] and is associated with a cost of reduced locomotor performance [64]. The functional interactions and relationships between individual newt toxicity and effects on individual snakes have been worked out in detail [27,58–63,65,66].

Both toxicity of newts and resistance of snakes vary geographically [28,60,62,63]. Where newts are absent or nontoxic, T. sirtalis are not resistant to TTX [28,62]. Elevated TTX resistance in western T. sirtalis is clearly derived, reaching levels 10–1,000 times that of other members of the genus in some populations [65]. Population differences in resistance are correlated with functionally important differences in amino acid sequences of skeletal muscle sodium channels [59]. Although considerable effort has been devoted to understanding the evolution of geographic and genetic patterns of TTX resistance in snakes, similar information regarding variation in newt toxicity is lacking. Data from only a few localities in the Pacific Northwest of North America suggest tight matching between prey and predator phenotypes [60], but a comprehensive survey of newt toxicity has not been previously conducted.

## Results

### Geographic Variation in Toxicity and Resistance

**Figure 2:**
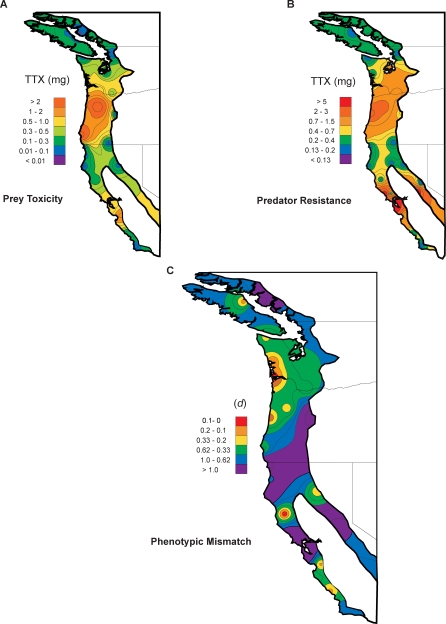
The Interpolated Geographic Distribution of Phenotypes and Phenotypic matching for Newts and Snakes in Western North America The interpolated geographic distribution of (A) prey toxicity, (B) predator resistance, and (C) the degree of phenotypic matching of these traits shown as isocline maps across the geographic range of sympatry in western North America. (A) Toxicity of prey shown as the total amount of tetrodotoxin (TTX) (mg) expected in the skin of an average adult animal based on the toxicity of 28 populations of *Taricha* newts in Figure 1. (B) Predator resistance shown as the amount of TTX (mg) required to reduce an average adult female T. sirtalis to 50% of its baseline performance based on the 28 populations in Figure 1. (C) The degree of phenotypic matching plotted as *d*, which measures the deviation from estimated match, at each locality, of the phenotypic interface of coevolution (i.e.. TTX toxicity in newts and TTX resistance of co-occurring snakes). Note that general overall patterns of relative phenotype exaggeration (yellow, orange, and red in (A) and (B)) are generally similar for newts and snakes throughout their range of sympatry. The matching analysis (Figure 3, Figure 4) and (C) shows that similarly elevated phenotypes do not necessarily match functionally.

Mean total skin TTX levels of newts ranged from no detectable TTX to 4.69 mg/newt and differed among populations (Figure 1; ANOVA: *F*_28, 382_ = 20.38, *p* < 0.0001). Across this phenotypic range, newt toxin levels were closely correlated with the resistance of sympatric snakes (Spearman ranked correlation, ρ = 0.71, *p* < 0.0001). Geographically, regions of highest newt toxicity also corresponded to regions of highest snake resistance, with extreme values of both traits found in the Willamette Valley of Oregon and the San Francisco Bay Area of California (Figure 1). Isocline maps of newt toxicity and snake resistance show generally similar spatial patterns of phenotypic variation (Figure 2A and 2B).

**Figure 3:**
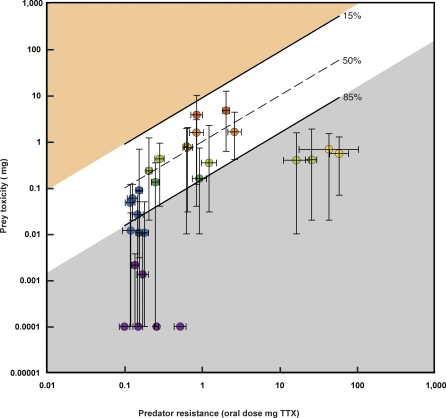
Mismatch of Predator and Prey Phenotypes at 28 Localities of Newts and Snakes from Western North America The functional relationship of average adult newt total skin toxicity (in mg TTX, log scale) is plotted against the oral dose of TTX (in mg, log scale) required to reduce the speed of an average adult female T. sirtalis to 50% of baseline speed post-ingestion against TTX (mg) for each locality. Individual points (circles) are colorized by average newt toxicity as in Figure 1. Vertical bars represent the full range of newt TTX values observed for each population; horizontal bars represent the 95% confidence interval for the oral dose 50% of the corresponding snake population. The 50% line (dashed) reflects the dose of TTX that would reduce a snake of given resistance to 50% of its performance (see Methods). The 15% and 85% lines (solid lines, calculated as best-fit regressions for each locality) delimit the range of functionally relevant TTX doses for snakes across the range of sampled localities (see Methods). Localities that fall outside the boundaries of these lines are considered mismatched. Below the 85% line (gray) are values of the phenotypic interface wherein garter snakes can consume co-occurring newts with no reduction in performance or fitness. Above the line (orange where no localities are observed) is phenotypic space where toxicity is so high that any snake that ingested a newt would be completely incapacitated or killed.

**Figure 4:**
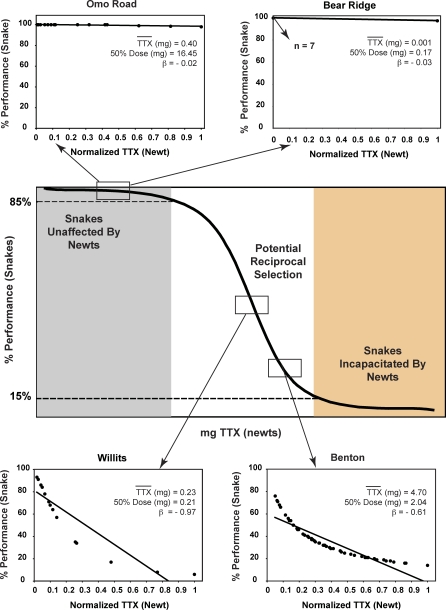
Representative Interaction Gradients for Localities across the Range of Mismatches The solid line s-shaped curve is the generalized dose response curve relating whole-snake resistance in snakes to quantity of TTX in mg. The position of this curve on the *x*-axis (TTX level) varies among localities (thus the lack of scale). The shaded regions correspond to the zones of mismatch evaluated based on phenotypic distributions of predator and prey phenotypes. From each zone (no localities were observed occupying the orange region), interaction gradients are shown for two example localities varying in newt toxicity. Interaction gradients are the linear regression of expected snake performance on individual whole-newt toxicities for a given locality, the slope (β) of which quantifies average potential selection due to the phenotypic interface. Zero or near-zero slope gradients indicate the absence of variance in fitness outcomes and therefore a lack of reciprocal selection. Zero slope localities illustrated include one with very low TTX (Bear Ridge, California), and one with intermediate TTX levels (Omo Road, California). Positive slope localities include one with intermediate (Willits, California) and one with very high TTX levels (Benton, Oregon). The full complement of interaction gradients can be found in Figure S1 and are summarized in Table S1.

Despite the overall spatial concordance of predator and prey phenotypes, an analysis of functional interaction reveals that over one-third of the localities sampled may qualify as ecological mismatches (Figure 3, Table 1, and Figure S1). The isocline map of the degree of mismatch, *d*, indicates that most of the geographic range of the newt–snake interaction is best characterized as mismatched and that regions of close ecological match are small and spatially restricted (Figure 2C, yellow to red areas). Localities where phenotypes are closely matched do not uniformly coincide with areas of elevated predator and prey phenotypes, but instead, include both ends of the phenotypic distributions of newts and snakes (Figures 1 and 2 and Table 1).

**Table 1:**
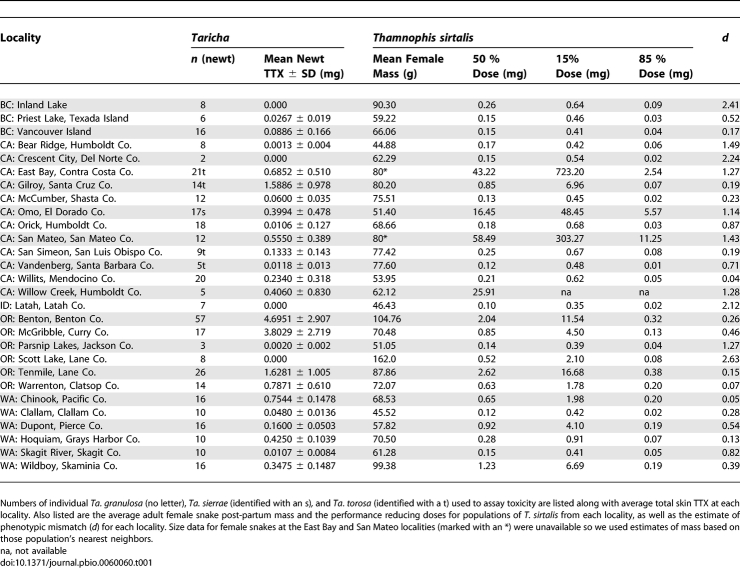
List of All Localities of Sympatric Newts and Snakes

### Phenotypic Mismatches

The observed levels of phenotypic mismatch ranged from near zero to *d* > 2.5 (Table 1). Values of *d* > 0.6 indicate populations that lay outside the 15% and 85% lines in Figure 3 and values of *d* < 0.6 indicate populations that fell between the 15% and 85% lines. Estimates of 0 < |*d*| < 0.6 indicate that localities lie within the zone of potentially experiencing reciprocal selection. In every case of mismatch (ten localities), predator resistance was much greater than the effective level of toxicity of local prey (Figure 3, gray zone, Table 1, and Figure S1A).

Mismatches included six populations of newts (Parsnip Lake, Oregon; Bear Ridge, California; Inland Lake, British Columbia; Crescent City, California; Latah, Idaho; and Scott Lake, Oregon) with little or no TTX (Figure 3, purple symbols in gray zone, Table 1, and Figure S1A). Snake populations at the same localities all fall in the lowest level of TTX resistance for garter snakes, wherein ingestion of ≈0.1–0.5 mg of TTX would reduce performance to 50%. This level of resistance is equivalent to the ancestral level of TTX resistance for the genus *Thamnophis* (50% dose ≈ 0.11 mg), including mostly species that have never coevolved with tetrodotoxic newts [65]. Four additional localities not explicitly recognized as mismatched (Skagit River, Washington; Orick, California; Priest Lake, British Columbia; and Vandenburg, California), also exhibited predator resistance greater than toxicity of local prey as well as reduced levels of resistance and toxicity (Figure 3, blue symbols in gray zone and one blue symbol in the nonshaded zone, Table 1, and Figure S1B). These nearly mismatched localities had phenotypic distributions of newt toxicity wherein a small reduction in the performance of local snakes could only result from ingestion of the most toxic newts present. We did not observe a single case where prey levels were greater than comparable predator abilities.

The other four mismatches include four populations of moderately to highly toxic newts (San Mateo, California; East Bay, California; Willow Creek, California; and Omo, California) that co-occur with the most resistant snake populations known (Figure 3, light green and yellow symbols in gray zone, Table 1, and Figure S1A). All of these localities occur within two geographic regions (the Bay Area of California and central Sierra Nevada) and include three species of *Taricha* (*Ta*. *torosa* and *Ta*. *granulosa* in the Bay Area and *Ta*. *sierrae* in the central Sierra Nevada) and likely two lineages of T. sirtalis [67]. As with the other mismatched populations, snakes in these localities can ingest sympatric newts with no reduction in performance or fitness consequence (Figure 4 and Figure S1A), however both newt and snake phenotypes are highly elevated, compared with ancestral conditions and conspecific populations (Figures 1, 2A, 2B, and 3, and Table 1).

Population-specific interaction gradients confirm the interpretation of mismatch across the interaction (Figure 4, Figure S1, and Table S1). Regressions of the predicted performance of snakes after ingestion of co-occurring newts indicate that TTX levels observed in newts at mismatched localities do not have variable effects (Figure 4, Figure S1A, and Table S1). Resulting interaction gradients in these mismatched populations have an average slope that is substantially lower that that seen in matched populations, and no differences in expected fitness are associated with variation in either TTX levels and TTX resistance at these localities (Figure 4, Figure S1, and Table S1). Interaction gradient slopes (β) of the four nearly mismatched localities were an order of magnitude greater than in mismatched populations, but much less than in matched localities, suggesting that these nearly matched localities experience reduced potential for selection relative to other matched populations, but greater than our mismatched localities (Figure S1 and Table S1).

## Discussion

Geographic patterns of newt toxicity and snake resistance illustrate the complexity of coevolutionary conditions that can span an extant species interaction. At first glance, levels of prey defense and predator exploitative ability appear to be generally correlated across broad geographic and phenotypic ranges (Figures 1and 2). Functional evaluation of trait distributions, however, shows that over one-third of sampled localities represent substantial mismatch in predator and prey abilities, including regions with extreme phenotypic values for both species (Figures 1–3). In many cases, these mismatches are so great as to obviate any current reciprocal selection (Figure 4 and Figure S1). In ten localities, predators are sufficiently resistant to escape selection due to prey toxicity, but prey are never too toxic to be successfully ingested by local predators. The pattern suggests a dynamic in which interacting species experience reciprocal selection, driving escalation of both prey and predator phenotypes in an arms-race style interaction. In some cases, predators have “escaped” this race by evolving extreme resistance to prey toxins, but the converse escape by prey is not observed. Other populations of predators and prey appear not to have entered the arms race at all.

### Mismatches at the Phenotypic Interface of Coevolution

Our results suggest, contrary to previous analyses [60], that extreme trait mismatches are not uncommon in this predator–prey system. However, the absence of localities in which newt toxicity was high enough to kill or disable any sympatric snake suggests that it is possible for the predator, but not the prey, to evolutionarily escape the reciprocal selection of the arms race. This directional asymmetry appears to contradict theoretical predictions arising from equilibrium theory [68], as well as the so-called “Life-Dinner Principle” (i.e., that prey experience stronger selection than predators in an arms race) [69], which predict that arms-race coevolution should favor defensive adaptations in prey over offensive adaptations in predators. This pattern may reflect a reversal in selective inequity as predicted for systems with deadly prey [70], or it may be particular to the unique biology of the newt–garter snake interaction.

The adaptive changes in resistance and toxicity are mediated not only by the strength of selection, but also by the genetic architecture of the traits at the interface. There is reason to expect adaptive changes in mechanisms of TTX resistance to proceed in a less-than-gradual fashion. If phenotypic changes in resistance are due to one or a few genes, then fixation of such genes in snake populations could be rapid and lead to phenotypic mismatches in one (or few) evolutionary step(s). Much of the variation in TTX resistance in T. sirtalis results from the expression of TTX-resistant voltage-gated sodium channels in skeletal muscle [58,59]. Resistance in these sodium channels is conferred by a small number of nucleotide substitutions in the TTX binding site [59]. The extreme resistance in at least one of these mismatched snake populations (Willow Creek, California) results from the substitution of a single amino acid [59]. Rapid fixation of such a simple mutation could explain how some populations of predators have escaped the arms race with prey. On the prey side, little is known about the basis of differences in TTX toxicity in newts, but some [52,71,72] have suggested that constraints on toxicity due to limited exogenous factors (e.g., environmentally derived precursors of TTX) may be one factor allowing predators to outpace prey in the arms race. However, the extreme levels of toxicity found in some newt populations demonstrate that elevated levels of the trait are possible within *Taricha*.

Our results indicate that geographic regions of phenotypic escalation are not necessarily congruous with coevolutionary hot spots. Coevolutionary hot spots (where reciprocal selection is intense) and cold spots (where selection is absent) are defined on the nature of the interaction rather than the level of the phenotype [1,2,4,27,60]. Our data reveal that current cold spots exist at localities with upper and lower extremes of phenotypes in both predator and prey (Figures 1–3). These results contradict earlier assessments of the geographic mosaic of coevolutionary hot spots in this system, which assumed that elevated predator phenotypes coincided with intense coevolution [60]. Similarly, reciprocal selection is possible at localities previously identified as cold spots (e.g., Vancouver Island, British Columbia) where phenotype distributions overlap and appear well matched despite the low levels of prey toxicity and predator resistance (Figure 1). Despite the fact that both predator and prey phenotypes show similar geographic patterns of escalation, the distribution of phenotype mismatch (i.e., cold spots) is not concordant (Figure 2). Coevolutionary hot spots may not be unequivocally assessed on phenotypic data alone, but at least the potential for reciprocal selection is observed across the range of phenotypic values in both taxa.

### Inferring Coevolutionary Dynamics

The observed pattern of trait mismatches among localities suggests a general arms-race dynamic for the process of predator–prey coevolution between *Thamnophis* and *Taricha*. The majority of localities occupy a broad band of phenotypic values within which potential reciprocal selection might occur (Figure 3). This zone of possible matching includes linearly increasing values of both newt toxicity and snake resistance that range from ancestral levels and increase several orders of magnitude, consistent with a counter-escalating arms-race dynamic in which pairs of populations experience reciprocal selection and evolve ever-increasing trait values [1,2,37,69]. This phenotypic zone includes multiple lineages of *Taricha* as well as at least two lineages of snakes that have evolved extreme levels of TTX resistance, suggesting that escalating dynamics have occurred multiple times during this evolutionary interaction [60,67,73,74]. Because many factors might ameliorate reciprocal selection at these localities, it is not possible to be certain that each of these localities represents a currently coevolving pair of populations. One clear and testable prediction from this interpretation is that older interactions should represent the populations with elevated phenotypes if average realized selection and the genetic architecture of toxicity and resistance are stable across localities.

Mismatched localities fall into two distinct groups that likely have different explanations and implications. At one end of the phenotypic distribution are the four populations of moderately to highly toxic newts that co-occur with the most resistant snake populations known (Figures 1 and 3, light green and yellow symbols in gray zone, and Table 1). As with other mismatched populations, snakes in these localities can ingest sympatric newts with no or little reduction in performance or fitness consequence, however both newt and snake phenotypes are highly elevated compared to ancestral conditions and conspecific populations. This pattern suggests that these localities have undergone arms-race coevolution, but that predators have escaped the arms race through the rapid evolution of extreme TTX resistance (see above). The extreme levels of TTX present at other localities (e.g. Benton, Oregon ) suggest that there does not appear to be a physiological limit to toxicity that explains these mismatches. These localities occur in nearby geographic regions (the Bay Area of California and the central Sierra Nevada; Figure 1) and involve different species of *Taricha* (*Ta. granulosa* and *Ta. torosa* in the Bay Area, and *Ta. sierrae* in the Sierra Nevada) [75]. Phylogeographic evidence suggests these represent two related groups of snake populations [67], indicating that escape from the arms race has occurred once or possibly twice in this fashion.

At the opposite end of the phenotypic distribution, we see mismatched localities from multiple lineages that appear never to have engaged in the arms race (Figures 1 and 3, purple symbols in gray zone). Population-specific interaction gradients (Table S1 and Figure S1A) and values of *d* (Figure 3 and Table 1) confirm that the opportunity for reciprocal selection in these localities is negligible. Both prey and predator traits at these localities appear to be close to estimated ancestral levels, wherein snakes have the slight ecological advantage of some predisposition to TTX resistance [60]. Average TTX levels in these newt populations range from 0 (or below our measurable lower limit of 0.0001 mg) to a high of around 0.002 mg (Parsnip, Oregon). This level of TTX is at or below the concentrations detected in related salamandrid species. In *Notophthalmus*, the sister genus to *Taricha*, reported levels of TTX range from 0 to a high of ≈0.06 mg per animal (estimated from [76]). In Cynops pyrrohgaster, an Asian TTX-bearing newt, typical whole-animal TTX levels range from 0–0.2 mg, with most population means ≈0.002 mg (estimated from [77]). The highest reported TTX level in the European newt genus *Triturus* (*sensu lato*) is 0.017 mg TTX (*Tr. cristatus*), with TTX levels in other species of *Triturus* an order of magnitude lower [78]. These comparative data suggest that mismatched localities at the low end of the phenotypic distribution have not engaged in counter-escalating coevolution. Alternatively, these populations may have been coevolving in the past, but once reciprocal selection was alleviated, costs of toxicity and resistance drove levels of both traits back to reduced levels. The four nearly mismatched localities (Figure 3, blue symbols in gray zone and one blue symbol in nonshaded zone) suggest that populations in this lower range can move from disengaged to engaged or that the process may be cyclical. Multiple snake and newt lineages are represented at the localities with unelevated phenotypes, suggesting that the phenomenon is not merely a phylogenetic artifact [67,73–75].

The apparent dynamic of arms-race coevolution in the newt–snake system, then, includes three or more stages. First, we see localities with low levels of traits at the phenotypic interface. Some of these localities, for reasons not yet clear, do not experience reciprocal selection and appear never to have engaged in the arms race. All of these localities involve predators able to subdue toxic prey without ill effect, suggesting that if newt toxicity rose in these populations, reciprocal selection would follow. As populations of newts gain toxicity (through mutation, migration from more toxic locales, or some exogenous influences), counter-escalation ensues and can lead to up to three–order-of-magnitude increases in traits. Initial increases in toxicity might be promoted by selection from interactions with other species as in other systems [3,17,22], including predators on early life stages [50]. Some localities (e.g., Benton, Oregon) seem to persist in this escalation zone, while others (e.g., Omo, California) escape from the arms race due to rapid evolution of extreme resistance through simple genetic mechanisms. Such adaptive changes suspend reciprocal selection, and no counter escalation follows. The next step for these populations is unclear. If costs to either resistance or toxicity are high enough, we might expect to see such escaped populations eventually lose phenotypic value and return to the lower left of Figure 3, resulting in de-escalation and a long-term cyclical dynamic. This scenario is plausible and has been suggested as an important dynamic in the chemically mediated coevolution between parsnip webworms and their host plant [79]. However, de-escalation is not supported by the observed patterns in the newt–snake interaction, which do not reveal mismatched localities with intermediate levels of resistance or toxicity.

## Methods

### Geographic sampling.

We sampled a total of 383 newts from 28 localities co-occurring with populations of garter snakes for which TTX resistance has been described [60]. This sampling regime included most of the geographic range of this interaction and included localities from the central coast of British Columbia to the central coast region of California (Figure 1). The number of individuals sampled for each locality ranged from two to a maximum of 57 (Table 1). Only sexually mature animals were assayed in order to minimize variation in toxin levels associated with ontogeny. We included both males and females in our analysis; sex ratios of specimens varied among localities. Although earlier work suggested that there might be minor gender differences in toxicity of *Ta*. *granulosa* [55], we detected no such differences in our data set (ANOVA: *F*_1, 370_ = 3.26, *P* = 0.0719). We sampled populations of *Ta. granulosa*, *Ta*. *torosa*, and *Ta*. *sierrae*. Because average toxicities of *Ta*. *torosa* and *Ta. sierrae* populations were completely within the range of *Ta*. *granulosa* populations, we included all three species in a single analysis for this study (Table 1).

### Model of the functional interaction between predator and prey.

We predicted and evaluated the distribution of expected performance outcomes for each population of snakes interacting with sympatric newts over the range of toxicity observed in newts from each given population. This model of the chemical ecology and physiology of the interaction is based on an extensive understanding of the functional interaction between newt toxicity and snake resistance [27,28,49,53,55,56,60–63,66]. For each locality, we estimated the toxicity (in mg of TTX) of newts, the doses of TTX (in mg) required to reduce performance of co-occurring snakes to 15%, 50%, and 85% of their baseline performance, and the degree of match or mismatch between newt and snake phenotypes (see below for details).

*Newt toxicity estimates and quantification of skin TTX levels.* The amount of TTX present in dorsal skin of individual newts was quantified with high-performance liquid chromatography–fluorescence detection, and estimates of total skin TTX (in mg) per animal were generated following previously published methods [28,49,50,53,55]. This methodology has been shown to be a highly repeatable and accurate method for measuring dorsal skin TTX levels [28,49,53,55] as well as for estimating the total skin TTX of individual animals [49].

*Quantification of TTX-resistance in* Th. sirtalis*.* Whole-animal resistance data (in mass-adjusted mouse units or MAMU) were taken from Brodie et al. [60], in which TTX-resistance was measured with a bioassay based on a reduction in organismal performance after an interperitoneal (IP) injection of TTX [60,62,63]. This bioassay provides a highly repeatable estimate of individual and population level differences in susceptibility to TTX that expresses resistance as a percentage of baseline locomotor performance. A measure of 50% resistance means that an individual (or population on average) could crawl at 50% of its baseline speed after an injection of a given amount of TTX. TTX-resistance estimates used here are based on data from a total of 2,449 snakes from 269 families from 28 populations. We used these published dose-response curves to interpolate the average 15%, 50%, and 85% IP resistance doses (in MAMU) for each locality.

*Comparing TTX toxicity in newts with TTX resistance in* Th. sirtalis. Because absolute levels of TTX resistance in snakes (i.e., doses in mg rather than in MAMU) are related to size [60,61,66], we adjusted population average TTX resistance with respect to post-partum female mass for each population. Adult females are the largest size class in a given population and therefore are the most likely to prey on newts. Additionally, because of asymptotic growth curves in snakes, adult females represent the best size class to compare across populations. The 15%, 50%, and 85% IP doses (in mg) of TTX for adult post-partum female snakes at each population of *Th*. *sirtalis* were thus estimated using the average mass of this demographic group at each locality (Table 1). In the case of one locality, East Bay, size data were unavailable and we used an estimate of the average female mass based on its nearest geographic neighbors. Because 1 MAMU = 0.01429 μg TTX per gram of snake [60,62,63,66,80], the IP dose of TTX (in mg) required to reduce performance to a given amount (e.g., 50%) for an adult female snake at any given locality is estimated as:

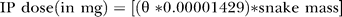
where θ is the performance reduction dose of interest (e.g., 15%, 50%, or 85% in MAMU) and snake mass is the mean post-partum weight of female snakes from a given population. We modeled the effect of oral consumption of newts by snakes by converting the above IP doses to oral dose. The relationship between oral and IP doses of TTX is linear for *Th*. *sirtalis* (as well as other vertebrates; e.g., mice). At all levels of resistance and doses of TTX, the oral dose required to achieve the same effect as an IP dose is 40× [61]. We converted the IP 15%, 50%, and 85% resistance doses (in mg) to oral doses (in mg) by multiplying each dose by 40.

Modeling mismatch. We defined a functional mismatch if ecological interactions between individuals of sympatric species do not result in variable fitness consequences for either taxa (i.e., all predators are able to subdue all prey without impairment, or all prey able to repel or kill all predators). We defined a given locality as “matched” if a sympatric interaction could potentially result in variable fitness outcomes for one or both taxa. This outcome was conservatively judged to occur if the average performance reduction of a local snake ingesting any sympatric newt fell between 15% and 85% of normal crawl speed. The phenotypic space referred to as “matched” is more properly the region wherein potential reciprocal selection could occur between TTX toxicity and resistance. At performance levels <15%, snakes that ingest newts are fully immobilized or killed and newts escape [81], whereas at performance levels >85% snakes are unaffected and all captured newts die. We visualized match and mismatch at individual populations by plotting total skin TTX of newts against the size adjusted, oral 50% dose of snakes at each locality along with 15% and 85% dose model lines (see below) on a log scale (Figure 3). The actual range of newt phenotypes at each locality was used to illustrate the distribution of prey phenotypes. Because predator phenotypes are based on an estimated asymptotic function, it was not possible to plot them as range and we used the 95% confidence interval around each localities 50% as an estimate of phenotypic range. Our data included populations of newts that had no measurable TTX; as a result we transformed all values (TTX in newts and 50% doses in snakes) by adding 0.0001 mg to each value. This adjustment maintained the overall relationship between newt phenotypes and snakes phenotypes but allowed zero values to be plotted. The 15%, 50%, and 85% model lines were plotted using the absolute (i.e., in mg) estimates of the 15%, 50%, and 85% resistance doses (see above for details) for each locality.

*Quantification of phenotypic mismatch.* We calculated (*d*) as the orthogonal distance from the joint mean of each locality to the predicted 50% performance line (Figure 3). This estimate of distance *d* from the best match provides a quantification of the degree of mismatch at a given locality. An analogous approach has been used to evaluate arms-races between the sexes within species [37]. Although the choice of 50% to express this mismatch metric is somewhat arbitrary, the model of performance was robust and returned similar results for a range of (40% to 60%) of hypothetical matches. Because of the extreme range and nonlinearity of snake 50% doses and the presence of newt populations that had TTX levels below our detectable levels, we used log-transformed values of the following—(newt total skin TTX + 0.0001) and (snake 50% dose + 0.0001)—to calculate *d* (see above). This method uses the equation for estimating the shortest distance from a point to a line:

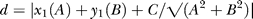
where *A* and *B* are the respective components of the slope and *C* is the intercept of the line. Our model assumes that the best functional match of newt and snake phenotypes at a given locality is one in which ingestion of an average newt by an average adult female snake will result in a reduction of that snake's crawl speed to 50% of baseline. This assumption results in the prediction that the model line describing perfect match is:

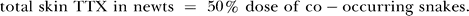



Thus the line describing perfect phenotypic match has a slope and intercept of 1 and 0 respectively, and *A* = 1, *B* = −1, and *C* = 0, and our estimate of *d* simplifies to:

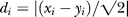
where *x_i_* = log (50 % dose + 0.0001) of snakes from a given locality, and *y_i_* = log (average total skin TTX + 0.0001) of co-occurring newts.

*Population-specific interaction gradients.* Interaction gradients were generated for each locality by estimating the performance reduction experienced by an average snake after ingesting any of the observed sympatric newts. Thus the gradients reflect the observed distributions of whole newt toxicity for each locality. Interaction gradients are estimated with simple linear regression (SNAKE PERFORMANCE = (NEWT TTX) * β + ERROR). to reveal the average slope of the fitness consequence analogous to directional selection gradients, regardless of the form of regression that best fits the data [82]. Snake performance values are calculated from population-specific dose-response curves (see above). For the purposes of plotting, we normalized newt TTX levels to range from 0–1, with the most toxic newts scaled to 1 for each locality.

### Visualizing geographic variation in phenotypes and mismatch.

*Phenotypic distributions and functional matching.* We used the quantitative estimate of mismatch *d* to visualize geographic patterns of mismatch. Isocline maps that included all sampled localities seen in Figure 1 were generated using inverse distance-weighted interpolation based on observed values (i.e., TTX levels, snake resistance, and *d*) and the latitude and longitude coordinates for each population. Because of nonlinearity in resistance values (see also [60]) oral 50% doses of >5 mg were entered as 5 mg. The function's power was set at two and the neighborhood at 500 km. Analyses were performed in ArcView GIS 3.3 with Spatial Analyst 2.0. Analysis of geographic patterns of TTX-resistance and justification for phenotype classes in snakes (Figure 2B) was performed as per [60]. We used multiple post-hoc comparisons to estimate phenotype classes for Figure 2A (newt total skin TTX). Populations with values of *d* > 0.6 (i.e., those that lie outside the range of the 15% and 85% dose lines and were considered mismatched) are colored in blue and purple (Figure 2C). Populations with values of *d* < 0.6 fell between the 15% and 85% lines and are colored in red, orange, yellow, and green (Figure 2C).

## Supporting Information

Figure S1 Interaction Gradients for 23 Sampled Localities across the Range of the Newt–Snake Interaction Each locality-specific interaction gradient is the linear regression of expected snake performance on individual whole-newt toxicities for that locality, the slope (β) of which quantifies average potential selection due to the phenotypic interface. For the purposes of plotting and comparison across localities, newt toxicities (on the *x*-axis) are normalized so that the most toxic animal from each population is represented as one and all others expressed as a ratio of that animal. Zero or near-zero slope gradients indicate the absence of variance in fitness outcomes and therefore a lack of reciprocal selection. Gradients are organized as mismatched (A) and potentially matched (B) and ordered within in each group by *d,* our quantitative estimate of mismatch (See Table 1 and Table S1). In four of the mismatched localities, the TTX levels of all sampled newts were below our measurable limit of detection or zero (Inland Lake, British Columbia; Crescent City, California; Latah, Idaho; and Scott Lake, Oregon; see Table 1). In these localities, the lack of variability in newt toxicity rendered it impossible to estimate β or plot an interaction gradient. In one additional population (Willow Creek, California), the extreme level and variation of resistance found in snakes prevented an accurate quantification of that population's dose-response curve (see [60]) preventing estimation of the locality specific interaction gradient for this site. Values of β as well as relevant statistical data associated with the gradients are summarized in Table S1.
(972 KB PDF)

Table S1 List of Interaction Gradient Statistics for All Localities Numbers of individual *Ta. granulosa* (no letter), *Ta. sierrae* (identified with an “s”), and *Ta*. *torosa* (identified with a “t”) used to assay toxicity are listed along with the average slope (β) of each population-specific interaction gradient. Also listed are *F* statistics, *p*-values, and 95% confidence intervals of (β) for each regression, as well as the estimate of phenotypic mismatch (*d*) for each locality. Localities marked with an asterisk (*) indicate populations in which the lack of variation in newt TTX levels prevented estimation of an interaction gradient or (β). Because of the extreme variation and level of resistance of Willow Creek snakes, no dose-response curve could be calculated (see [60]). At Bear Ridge, the bimodal distribution of newt toxicity prevented statistical assessment of β.
(88 KB DOC)

## Abbreviations

IP: interperitoneal
MAMU: mouse-adjusted mass unit
TTX: tetrodotoxin
