# Supplementary material for: Phenotypic Mismatches Reveal Escape from Arms-Race Coevolution
Source: PLoS Biol. 2008 Mar 11;6(3):e60. doi: 10.1371/journal.pbio.0060060 (PMC2265764; doi:10.1371/journal.pbio.0060060)

A. Mismatched Localities

Elevated Phenotypes

East Bay

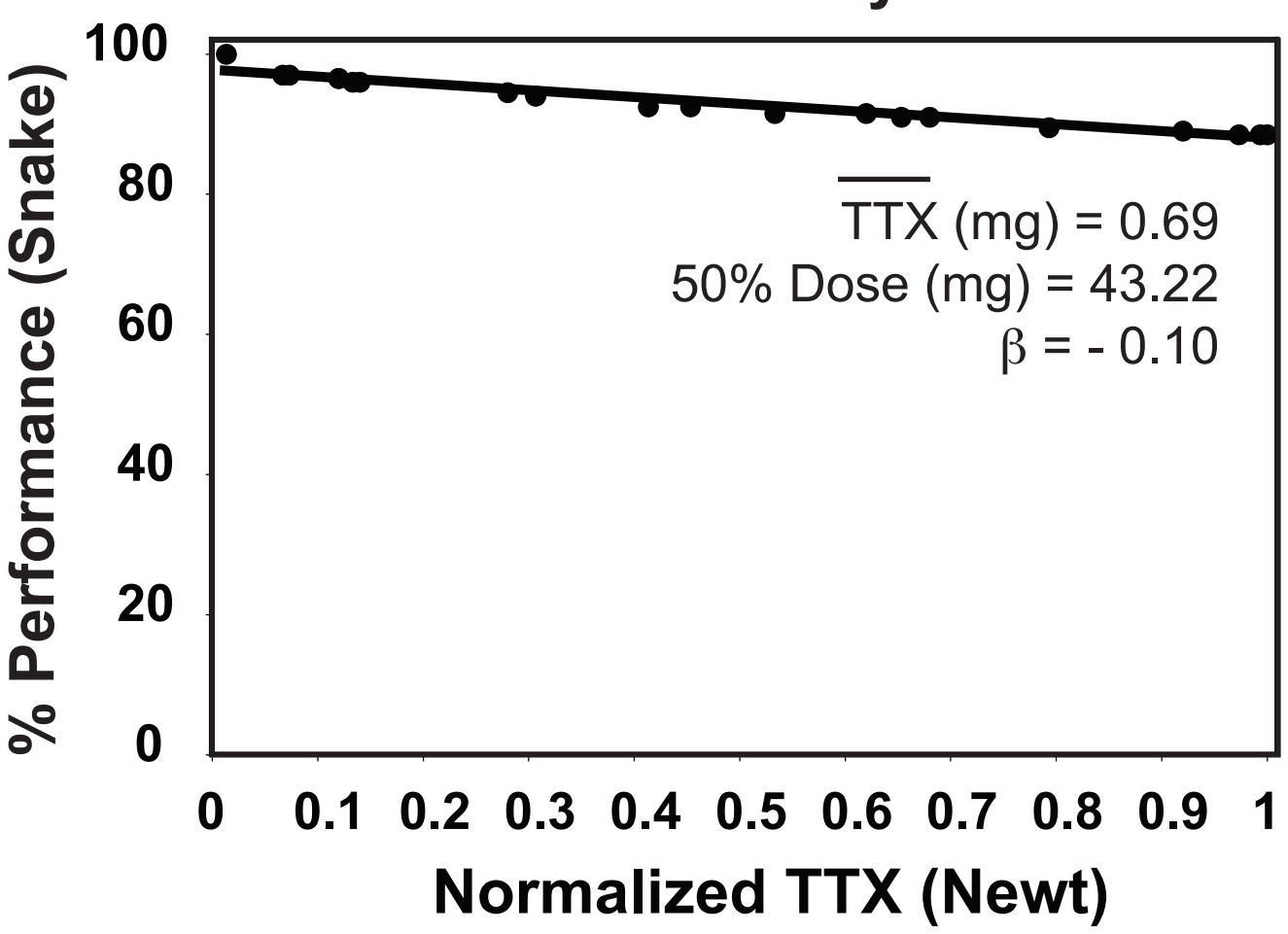

Omo Road

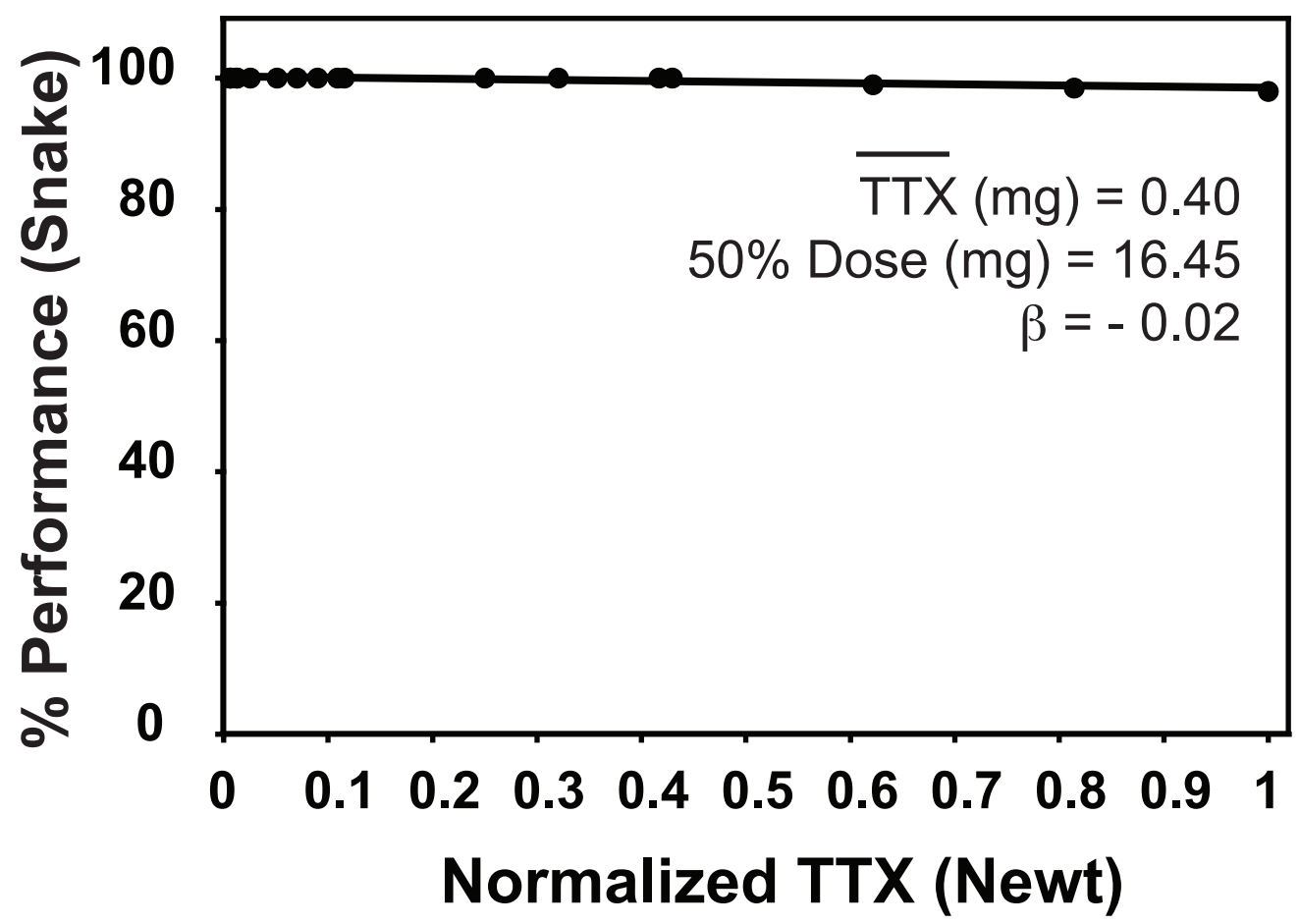

San Mateo

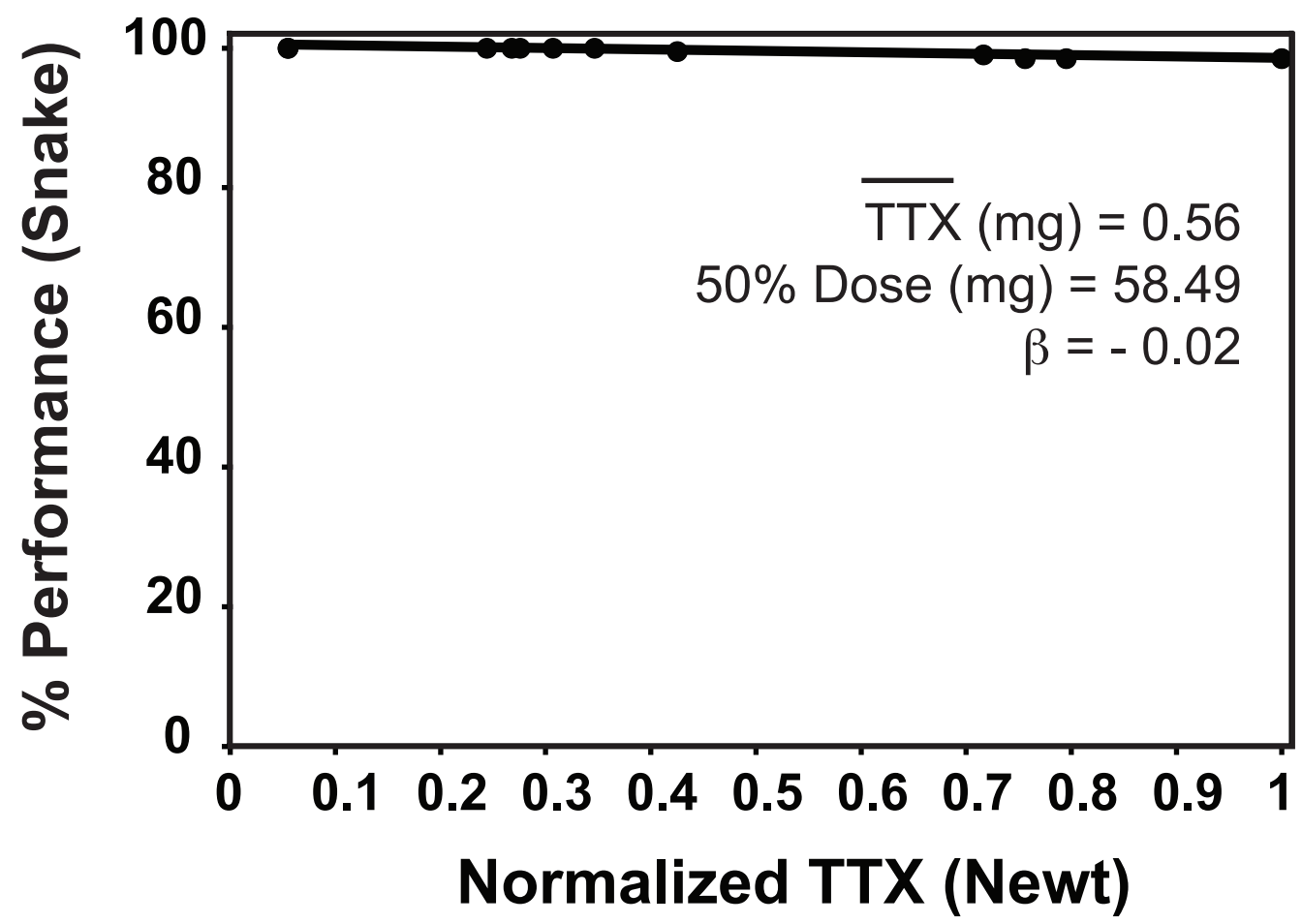

"Ancestral" Phenotypes

Parsnip Lake

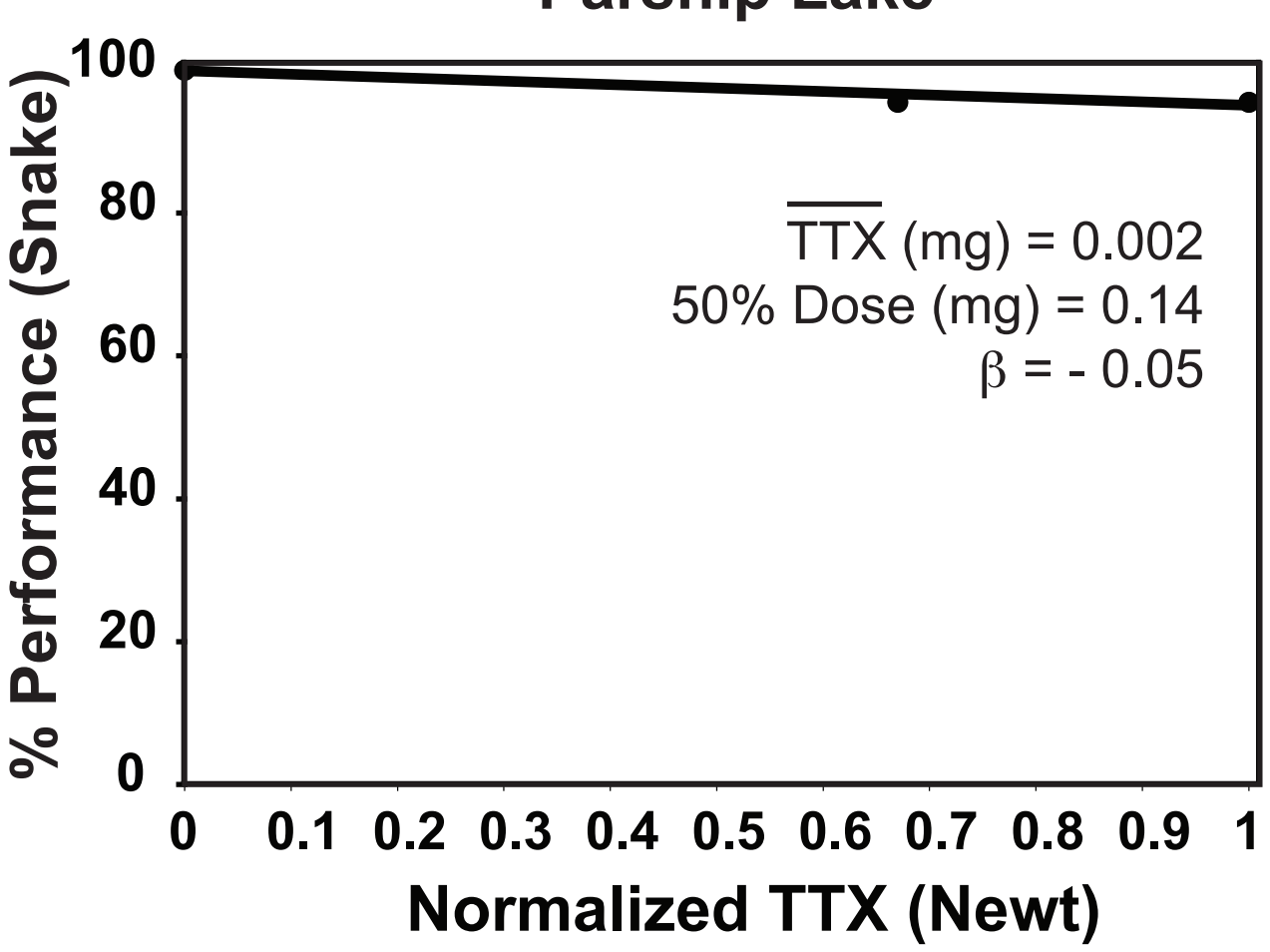

Bear Ridge

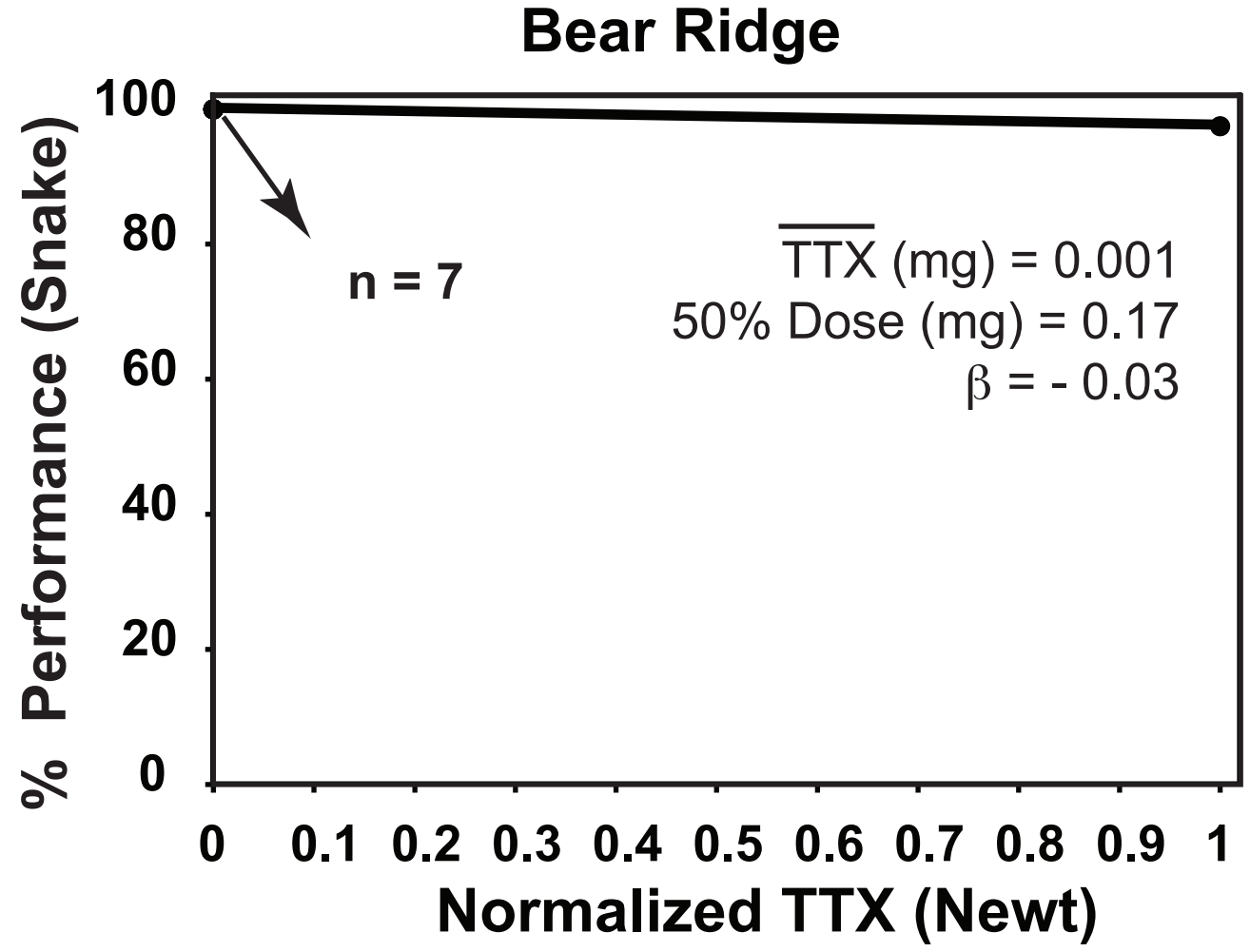

B. Potentially Matched Localities

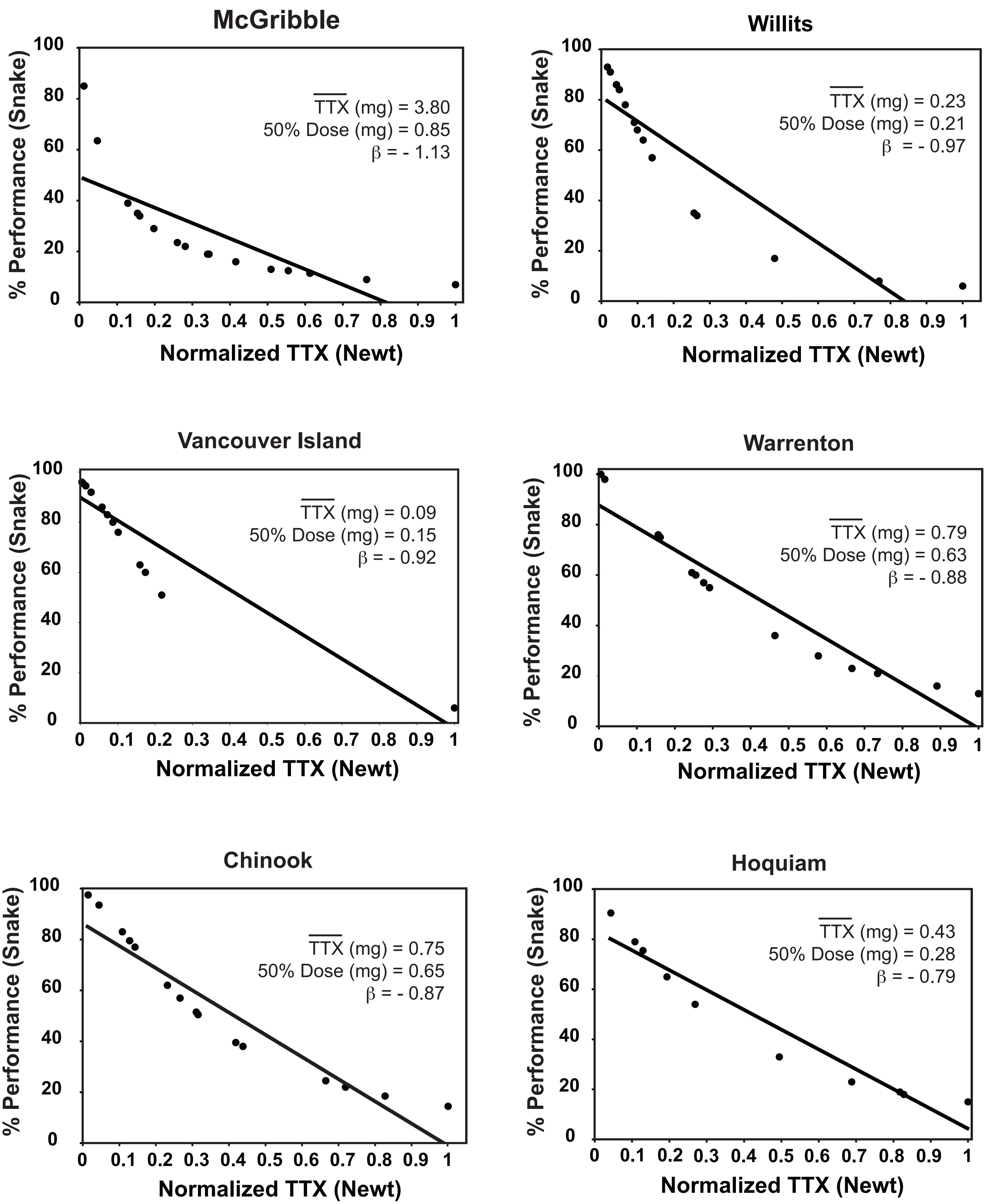

B. Potentially Matched Localities (cont.)

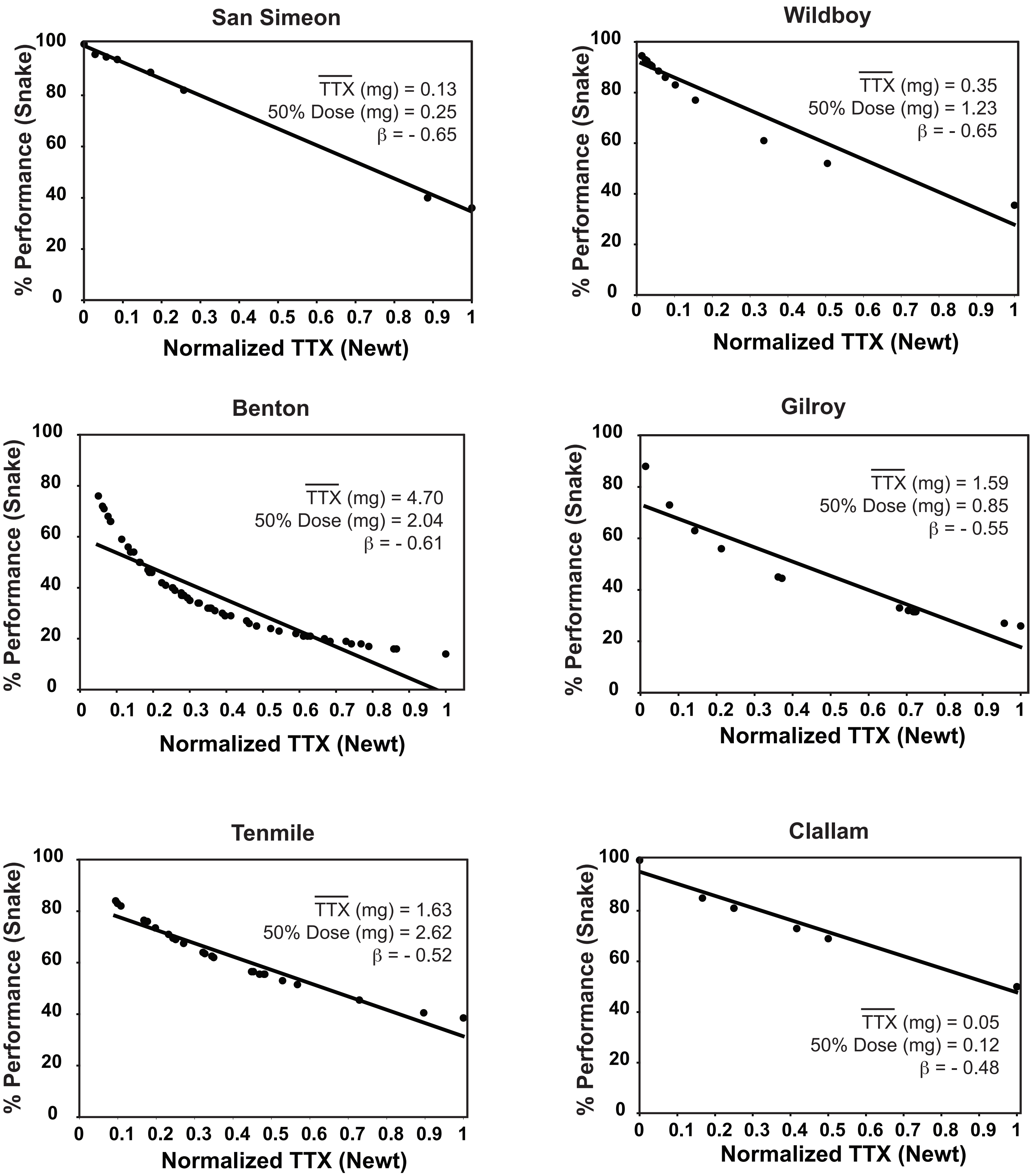

B. Potentially Matched Localities (cont.)

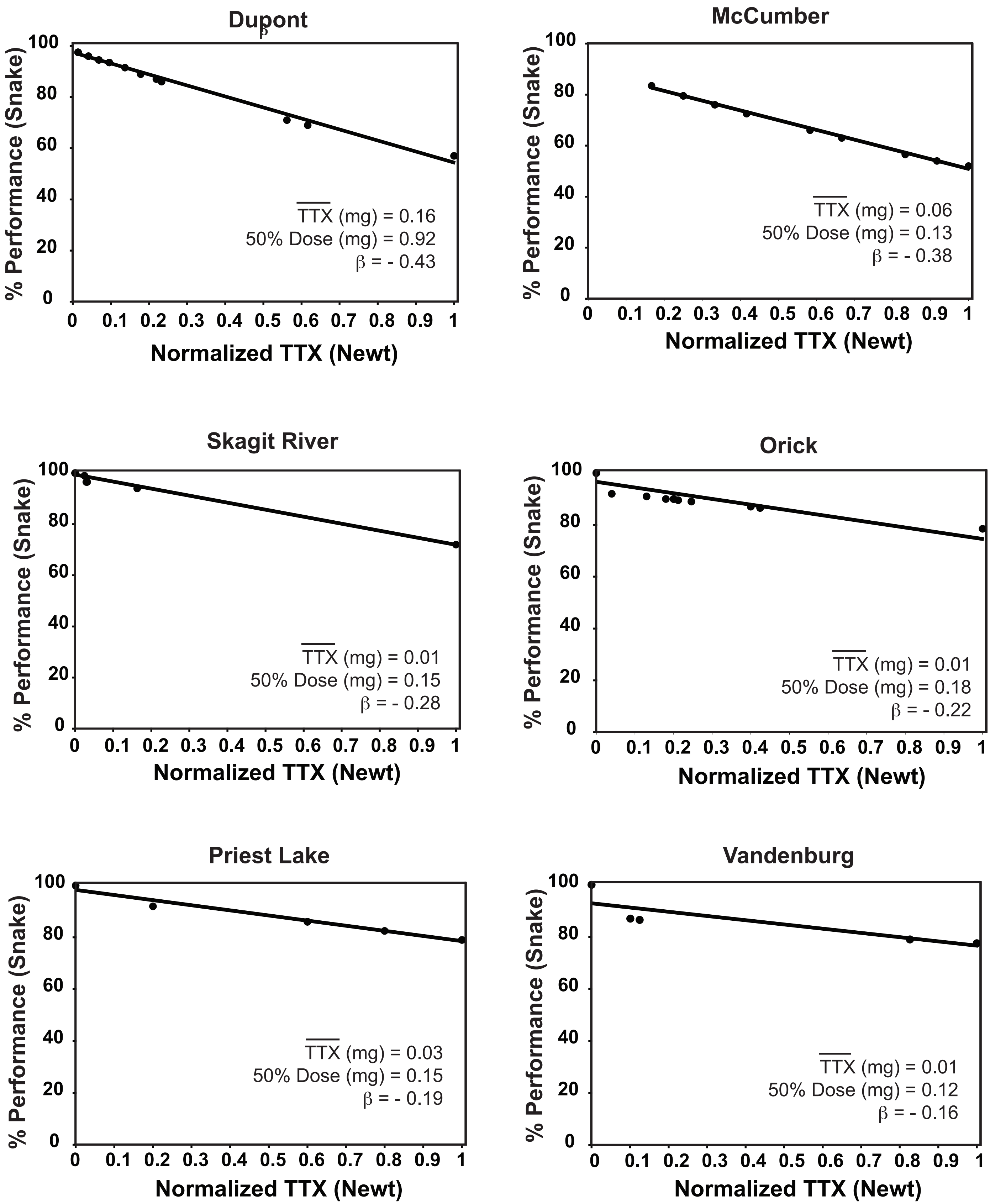

Supplement: Supplementary file 1 [file pbio.0060060.sg001.pdf]
