# Supplementary material for: Phenotypic Mismatches Reveal Escape from Arms-Race Coevolution
Source: PLoS Biol. 2008 Mar 11;6(3):e60. doi: 10.1371/journal.pbio.0060060 (PMC2265764; doi:10.1371/journal.pbio.0060060)
Supplement: Supplementary file 2 [file pbio.0060060.st001.doc]

**Table S1.**

| **Locality** | **n (newt)** | **β** | **F** | **p** | **95% CI (beta)** | | **d** |
| --- | --- | --- | --- | --- | --- | --- | --- |
|  |  |  |  |  | **lower** | **upper** |  |
| CA: Omo, El Dorado Co. | 17s | -0.018 ± 0.002 | 62 | < 0.001 | -0.022 | -0.013 | 1.14 |
| CA: San Mateo, San Mateo Co. | 12 | -0.021 ± 0.002 | 74 | < 0.001 | -0.026 | -0.015 | 1.43 |
| CA: Bear Ridge, Humboldt Co. | 8 | -0.03 ± na | na | na | na | | 1.49 |
| OR: Parsnip Lakes, Jackson Co. | 3 | -0.048 ± 0.016 | 9 (8.6) | 0.21 | -0.258 | 0.161 | 1.27 |
| CA: East Bay, Contra Costa Co. | 21t | -0.097 ± 0.005 | 374 | < 0.001 | -.0108 | -0.087 | 1.27 |
| CA: Vandenberg, Santa Barbara Co. | 5t | -0.163 ± 0.058 | 8 (7.5) | 0.068 | -0.349 | 0.023 | 0.71 |
| BC: Priest Lake, Texada Island | 6 | -0.197 ± 0.018 | 122 | < 0.001 | -0.247 | -0.148 | 0.52 |
| CA: Orick, Humboldt Co. | 18 | -0.220 ± 0.029 | 59 | < 0.001 | -0.281 | -0.159 | 0.87 |
| WA: Skagit River, Skagit Co. | 10 | -0.275 ± 0.013 | 480 | < 0.001 | -0.303 | -0.246 | 0.82 |
| CA: McCumber, Shasta Co. | 12 | -0.381± 0.006 | 3501 | < 0.001 | -0.396 | -0.367 | 0.23 |
| WA: Dupont, Pierce Co. | 16 | -0.431 ± 0.031 | 1414 | < 0.001 | -0.455 | -0.406 | 0.54 |
| WA: Clallam, Clallam Co. | 10 | -0.477 ± 0.031 | 231 | < 0.001 | -0.549 | -0.404 | 0.28 |
| OR: Tenmile, Lane Co. | 26 | -0.516 ± 0.028 | 345 | < 0.001 | -0.573 | -0.459 | 0.15 |
| CA: Gilroy, Santa Cruz Co. | 14t | -0.554 ± 0.058 | 90 | < 0.001 | -0.681 | -0.427 | 0.19 |
| OR: Benton, Benton Co. | 57 | -0.614 ± 0.040 | 235 | < 0.001 | -0.694 | -0.534 | 0.26 |
| WA: Wildboy, Skaminia Co. | 16 | -0.645 ± 0.045 | 208 | < 0.001 | -0.741 | -0.549 | 0.39 |
| CA: San Simeon, San Luis Obispo Co. | 9t | - 0.648 ± 0.012 | 3156 | < 0.001 | -0.675 | -0.621 | 0.19 |
| WA: Hoquiam, Grays Harbor Co. | 10 | -0.792 ± 0.074 | 116 | < 0.001 | -0.961 | -0.622 | 0.13 |
| WA: Chinook, Pacific Co. | 16 | -0.872 ± 0.078 | 126 | < 0.001 | -1.039 | -0.705 | 0.05 |
| OR: Warrenton, Clatsop Co. | 14 | -0.884 ± 0.076 | 135 | < 0.001 | -1.050 | -0.719 | 0.07 |
| BC: Vancouver Island | 16 | -0.920 ± 0.091 | 102 | < 0.001 | -1.115 | -0.072 | 0.17 |
| CA: Willits, Mendocino Co. | 20 | -0.965± 0.110 | 77 | < 0.001 | -1.196 | -0.734 | 0.04 |
| OR: McGribble, Curry Co. | 17 | -1.128 ± 0.182 | 38 | < 0.001 | -1.517 | -0.739 | 0.46 |
| BC: Inland Lake | 8 | * |  |  |  |  | 2.41 |
| CA: Crescent City, Del Norte Co. | 2 | * |  |  |  | | 2.24 |
| CA: Willow Creek, Humboldt Co. | 5 | na |  |  |  | | 1.28 |
| ID: Latah, Latah Co. | 7 | * |  |  |  | | 2.12 |
| OR: Scott Lake, Lane Co. | 8 | * |  |  |  | | 2.63 |
